# Encapsulating Peritoneal Sclerosis Improvement Following Conservative Treatment in a Child Undergoing Peritoneal Dialysis

**DOI:** 10.7759/cureus.77359

**Published:** 2025-01-13

**Authors:** John Dotis, Antonia Kondou, Vasiliki Karava, Nikoleta Printza

**Affiliations:** 1 Department of Pediatrics, Hippokration General Hospital/Aristotle University of Thessaloniki, Thessaloniki, GRC; 2 Department of Nephrology, Agia Sofia Children's Hospital, Athens, GRC

**Keywords:** child, conservative treatment, encapsulating peritoneal sclerosis, peritoneal dialysis, tamoxifen

## Abstract

Encapsulating peritoneal sclerosis (EPS) constitutes a rare but one of the most serious complications of long-term peritoneal dialysis (PD) in children. The role of abdominal computed tomography (CT) imaging in the early recognition and management of EPS is of great importance and should be performed promptly to establish diagnosis. This fact can provide an opportunity for conservative treatment with corticosteroids and tamoxifen, as it appears that early treatment can have beneficial effects. Based on the above, we present a five-year-old girl undergoing PD who developed EPS established by abdominal CT, who was withdrawn from the PD method but was successfully treated with a combination of prednisone and tamoxifen without the need for a surgical approach.

## Introduction

Encapsulating peritoneal sclerosis (EPS) is an extremely rare complication of long-term peritoneal dialysis (PD), characterized by persistent, intermittent, or recurrent adhesive bowel obstruction with features of encapsulation due to adhesions of a diffusely thickened peritoneum, as defined by the International Society of Peritoneal Dialysis [1]. The exact mechanisms explaining the reasons that people develop EPS have not been fully explored. However, it seems that a series of events, mainly including the chronic exposure of the peritoneum to PD fluids, could cause the activation of various inflammatory manifestations, ultimately leading to progressive peritoneal fibrosis, vasculopathy, and calcification [2]. In addition, the incidence of EPS probably increases with the length of time on PD [3]. Although there is limited data for children, the prevalence of EPS in European children on PD seems to be comparable with that of adult PD patients, but fortunately, mortality from pediatric EPS is significantly lower [2].

Rarely, some patients with EPS can be asymptomatic for a long time, but commonly, the clinical presentation is recurring abdominal pain and acute or subacute small bowel obstruction without any other concerning abnormalities in the patient's history [4]. Occasionally, patients can also present with anorexia, nausea, vomiting, or ascites, while an abdominal mass formed of the cocooned gut is palpable and has also been described [2,4]. Until recently, it seems that diagnosis is mostly made during surgical exploration. However, abdominal computed tomography (CT) imaging, as compared to other radiological methods, showed better specificity and sensitivity and might play an important diagnostic role in case of clinical suspicion [5].

The treatment of EPS is still controversial with surgical intervention, including resection of the thickened and limiting membrane with enterolysis being the most appropriate treatment modality in many cases. However, a combination of cessation of PD as well as the use of corticosteroids, tamoxifen, and immunosuppression seems to be a very promising alternative therapy [2,6,7]. Data on the application of conservative treatment in children with EPS are scarce. We hereby present a case of EPS in a child undergoing PD with the aim to emphasize that a high clinical suspicion is required for the early recognition of EPS in order to have the possibility for conservative treatment, as it appears that early treatment can have beneficial effects.

This article was previously presented as a meeting abstract/poster at the 56th European Society for Paediatric Nephrology (ESPN) Annual Meeting 2024 on September 24-27, 2024, in Valencia, Spain.

## Case presentation

We herein describe a case of a five-year-old girl who developed EPS during PD treatment. She initiated PD, in particular, the nocturnal intermittent PD (NIPD) program, from the age of 12 months due to polycystic kidney disease. From her past history, there were several PD-related infections, such as exit site infection (ESI) and peritonitis. Specifically, at the age of 18 months, she developed ESI due to methicillin-sensitive *Staphylococcus aureus*. Next, at the age of 24 months, she suffered a combined peritonitis plus ESI due to methicillin-resistant *Staphylococcus aureus*, resulting in the replacement of the Tenchoff catheter with a new one in a different position. Lastly, at the age of 54 months, she developed peritonitis due to *Pseudomonas aeruginosa* treated with prompt antimicrobial therapy. For all these four years, despite infections, she followed her NIPD program without any ultrafiltration problems.

Before the admission, she developed signs and symptoms consistent with peritonitis, such as cloudy peritoneal effluent, low-grade fever (38.0°C) for 12 hours, abdominal pain, and vomiting. Although biochemical investigation of the peritoneal effluent was indicative for peritonitis, multiple cultures were negative for any pathogen (Table 1).

**Table 1:** Biochemical and microbiologic investigation of the peritoneal effluent during hospitalization NA - not available *Based on the International Society for Peritoneal Dialysis (ISPD) guidelines, diagnosis of peritonitis is made when two of the three following criteria are met: 1) clinical features consistent with peritonitis (e.g., abdominal pain, cloudy dialysis effluent); 2) dialysis effluent white blood cell (WBC) count of >100 cells/μL or >0.1 × 109 cells/L (after a dwell time of at least 2 hours), with >50% polymorphonuclear leukocytes (PMNs); and 3) positive effluent culture [8]

Day (peritoneal effluent)*	White blood cell count (cell/μl)	Polymorphonuclear (PMN) cell ratio	Culture
1	760	77%	negative
5	150	52%	negative
10	1800	67%	negative
15	400	48%	negative
20	55	NA	NA
25	490	41%	negative
30	10	NA	NA

**Figure 1:**
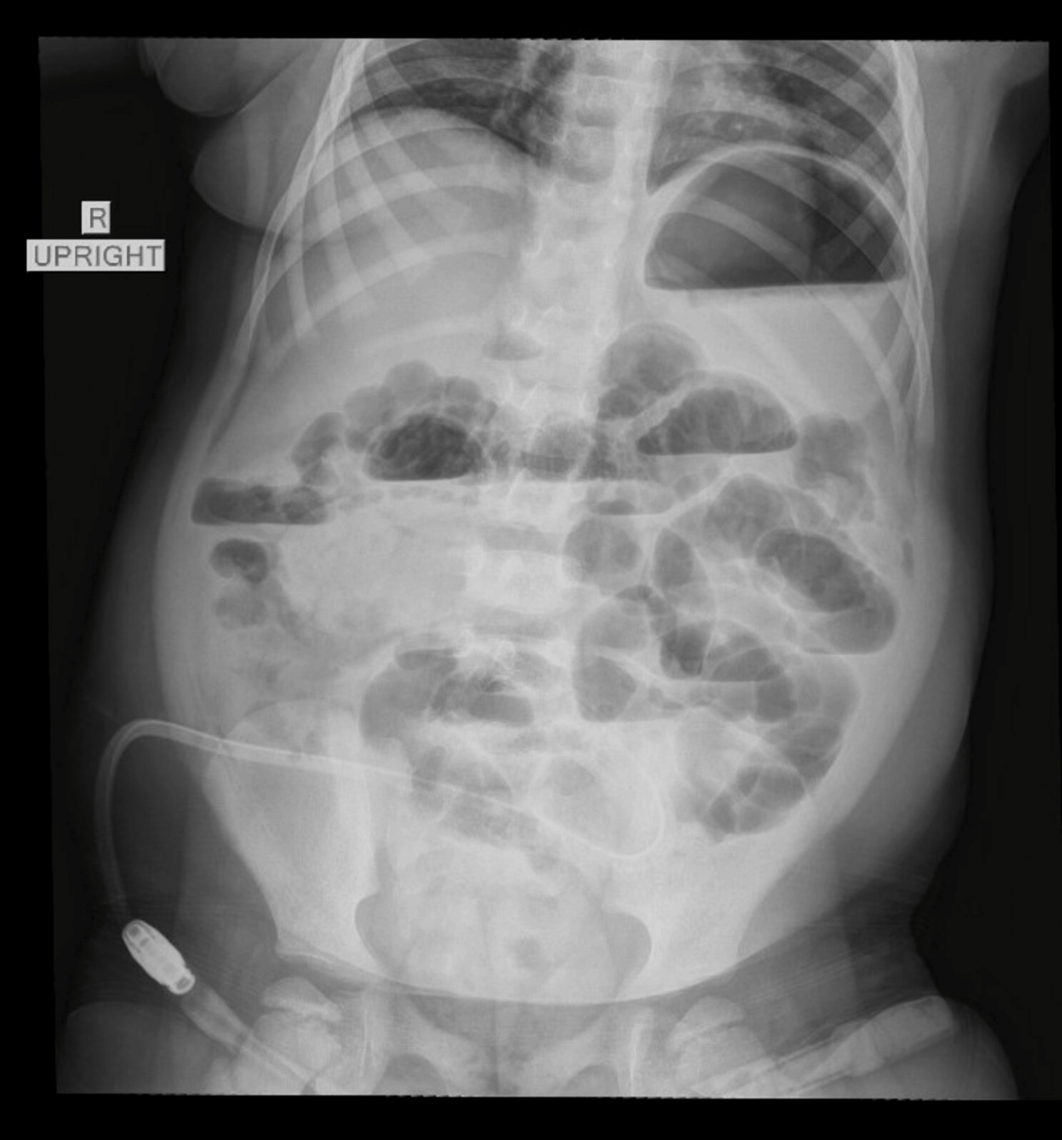
Abdominal X-ray showing multiple dilated loops of small bowel with air-fluid levels. The presence of a Tenkhoff catheter is visible.

Ultrafiltration insufficiency was observed, indicating low ultrafiltration capacity due to membrane dysfunction. A peritoneal equilibration test (PET) showed a deterioration of peritoneal dialysis treatment efficiency as compared to the previous one, six months earlier. A further imaging study was performed, that included an X-ray of the abdomen, showing multiple dilated loops of small bowel with air-fluid levels (Figure 1), while an abdominal ultrasound demonstrated, revealing an echogenic thickening of the bowel walls of uncertain etiology.

**Figure 2:**
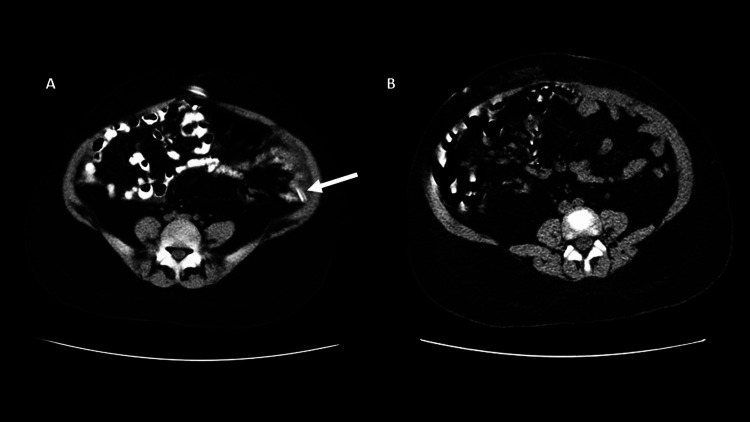
A) Abdominal axial contrast enhanced computed tomography imaging shows the smooth thickened peritoneal covering and numerous peritoneal calcification, bowel dilation with irregular thickening both findings indicating encapsulating sclerosing peritonitis (Tenkhoff catheter is indicated by the arrow). B) Abdominal axial contrast enhanced computed tomography imaging, 6 months later, shows an obvious improvement of the findings as compared to the previous exam, with a great reduction in the number of calcifications and partial recovery of the peritoneum and the bowel.

In addition, the patient underwent abdominal CT imaging; the findings were compatible with EPS (Figure 2A). A biopsy was not performed as the abdominal CT imaging findings were sufficiently illuminating and diagnostic. The patient was put immediately on therapy with tamoxifen 10 mg daily in addition to prednisone (1 mg/kg initial dose, progressively tapering to 0.25 mg/kg). PD was stopped while the Tenchoff catheter was removed, and the patient was switched to hemodialysis. Six months later, during a follow-up, the CT imaging of the abdomen revealed a partial resolution of the findings (Figure 2B) while treatment with tamoxifen was stopped, and a low dose of prednisone (0.25 mg/kg) was continued.

**Figure 3:**
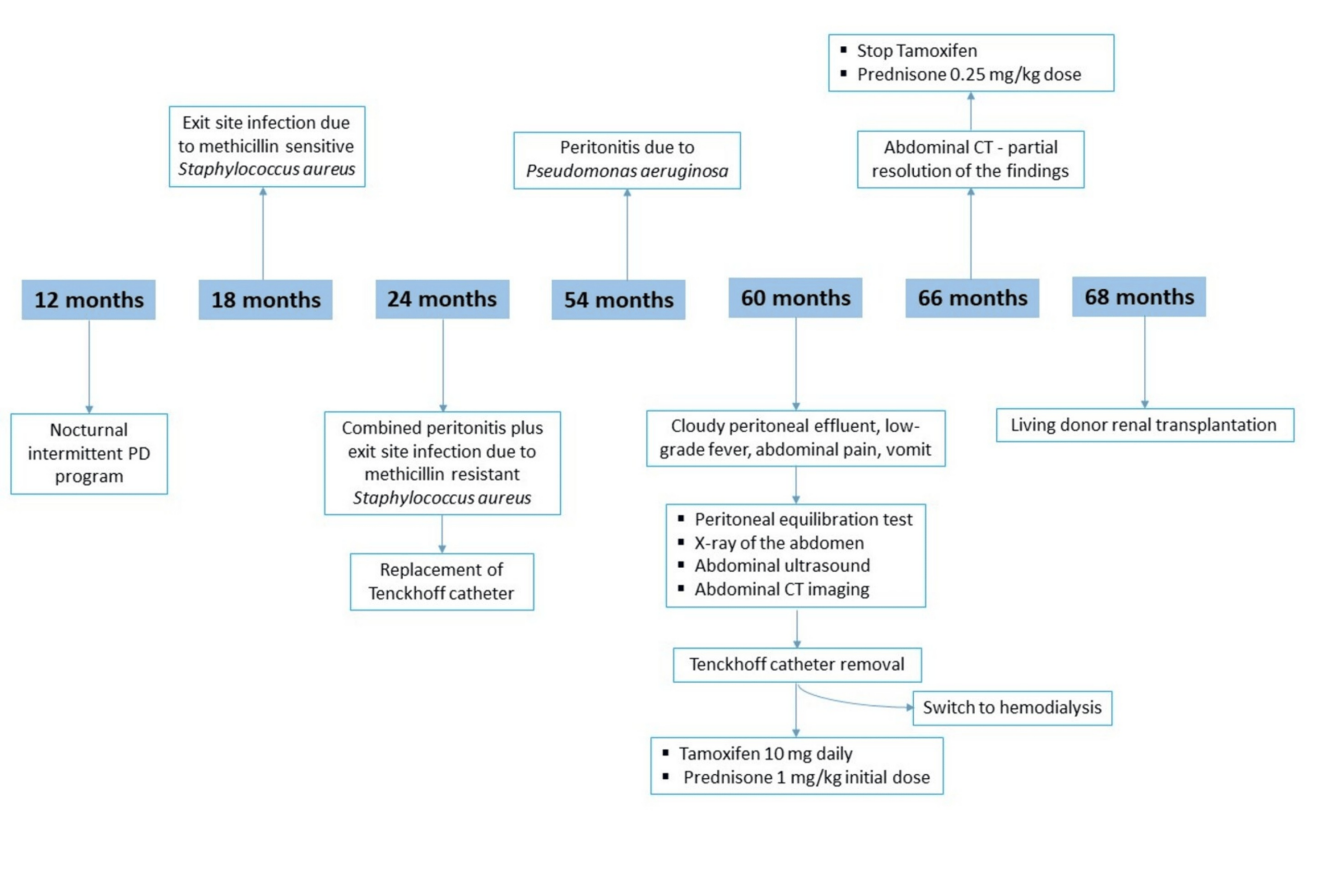
Diagnostic and treament timeline of the patient PD - peritoneal dialysis; CT - computed tomography

The patient underwent a living donor renal transplantation after eight months on hemodialysis. Now, 12 months after the transplantation, the patient has shown no recurrent symptoms of EPS, while her estimated glomerular filtration rate (eGFR) is normal and greater than 90 ml/min/1.73m2. A comprehensive diagnostic timeline that outlines the progression of symptoms, imaging studies, clinical decisions, and outcomes is presented in Figure 3.

## Discussion

EPS is a rare complication of PD that is associated with significant morbidity and mortality, resulting in withdrawal from the PD method. Although the data from children is limited, a survey from the European Paediatric DialysisWorking Group revealed that 1.5% of children with PD will suffer from EPS [2]. In the same study was found that 77% of children received immunosuppression with prednisolone, and 41% received tamoxifen. However, 64% of children were dealt with surgical management by partial/complete enterolysis or laparotomy for bowel perforation. Unfortunately, 13.6% of patients died due to complications of EPS, indicating the seriousness of this pathological entity [2]. At this point, with our case, we would like to emphasize that high clinical suspicion, with the help of abdominal CT as a diagnostic tool for early recognition of EPS, can lead to conservative treatment that seems ideal for such cases. In addition, our patient was stabilized with corticosteroids and tamoxifen with partial response, then underwent transplant relatively early, which might have played a more prominent role in 'curing' EPS. It might also be possible that EPS was diagnosed early, so the disease was not that severe, given the fact that she did not need any surgical intervention, which might explain her relatively good response to conservative treatment.

To the best of our knowledge by searching the English literature, only two cases of EPS in children or adolescents during PD treatment have been described in detail and were treated with tamoxifen, although in retrospective studies/case series, there are reports in pediatric patients without providing descriptive presentation of clinical findings, diagnostic method, and treatment. The first case was a 12-year-old boy on continuous ambulatory PD from age seven, suffering from numerous peritonitis episodes. He was admitted to the hospital with symptoms mimicking peritonitis; however, imaging studies, especially abdominal CT findings, were suggestive of EPS. Treatment with tamoxifen, in addition to prednisone, was initiated for three months, and his symptoms improved gradually over two months, continuing renal replacement therapy with hemodialysis. Eight months later, he underwent a renal transplantation [9]. The second case was an 18-year-old girl undergoing a PD program for seven years, having multiple peritonitis episodes during the years. The patient developed progressive abdominal distension, which led to the diagnosis of EPS based on abdominal CT findings, and was started treatment with corticosteroids and tamoxifen, continuing renal replacement therapy with hemodialysis. Two months after initiation of treatment for EPS, the patient received a kidney transplant, while tamoxifen had been used for 10 months [10]. Noticeable is the fact that neither of the two patients nor our patient required a biopsy or a surgical procedure to establish the diagnosis of EPS. In addition, all patients had a remarkable improvement in abdominal CT findings after treatment with tamoxifen and corticosteroids and were able to be transplanted without any problems at all and mostly without complications. Noticeable is the fact that in the survey of the European Paediatric Dialysis Working Group, constituting the largest review of pediatric EPS cases, out of 22 patients were reviewed, 19 received immunosuppression or tamoxifen [2]. Unfortunately, there was no further analysis for these cases.

It turns out that in most ESP cases, the diagnosis is made intraoperatively, although preoperative diagnosis can be achieved due to vigilance and a high level of clinical suspicion [11]. Because early clinical signs are usually nonspecific or may mimic peritonitis, the condition may not be recognized until the patient develops partial or total small bowel obstruction [2,12].

Taking in account the non-specificity of ESP clinical findings, imaging methods become a useful tool for an early and prompt diagnosis, directly contributing in the adoption of an appropriate treatment. In patients with ESP, abdominal CT imaging reveals aggregated and dilated loops of small bowel concentrated in an abdominal segment, involved by a thick membrane, with peritoneal thickening, ascites, localized fluid collections, and possible peritoneal calcifications [13]. Therefore, as compared with other imaging techniques, abdominal CT provides a comprehensive view and is characterized as the gold standard imaging modality for the detection of peritoneal abnormalities and encapsulation [13,14]. In addition, aids in making a differential diagnosis of peritoneal diseases and can lead to early identification of ESP.

The accumulating clinical experience with tamoxifen, especially in adults, is encouraging for the treatment of ESP [7]. Tamoxifen is thought to inhibit fibroblast transforming growth factor (TGF)-β1 production, thereby preventing TGF-β1-driven peritoneal thickening and fibrosis [15]. Based on its properties, it seems that it can play a determinant role in peritoneal restoration after ESP, but the data on children are limited [2,9,10]. Nevertheless, they are very encouraging, and either as a monotherapy, or as a combined treatment with low-dose corticosteroids, it can be decisive in the treatment of ESP. Corticosteroids are preferred during the inflammatory stage, while tamoxifen may be beneficial during the fibrotic stage. These speculations can be strengthened by the successful outcome of our patient. However, we suggest the need for larger studies or randomized controlled trials to validate the effectiveness of conservative treatment for pediatric EPS.

## Conclusions

In conclusion, EPS can be diagnosed by specific features of abdominal CT, making surgical procedures unnecessary under certain circumstances. This case report highlights the importance of EPS early diagnosis and initiation of conservative treatment with corticosteroid and tamoxifen, although randomized controlled trials of tamoxifen and corticosteroid use in EPS would be required. Early diagnosis, conservative treatment, and transplantation relatively early seem to be key points in such cases. This report will alert pediatric nephrologists to this rare but extremely serious complication of chronic PD and allow for early diagnosis and prompt treatment, avoiding surgery.

## References

[1] Kawaguchi Y, Kawanishi H, Mujais S, Topley N, Oreopoulos DG (2000). Encapsulating peritoneal sclerosis: definition, etiology, diagnosis, and treatment. International Society for Peritoneal Dialysis Ad Hoc Committee on Ultrafiltration Management in Peritoneal Dialysis. Perit Dial Int.

[2] Shroff R, Stefanidis CJ, Askiti V (2013). Encapsulating peritoneal sclerosis in children on chronic PD: a survey from the European Paediatric Dialysis Working Group. Nephrol Dial Transplant.

[3] Tseng CC, Chen JB, Wang IK (2018). Incidence and outcomes of encapsulating peritoneal sclerosis (EPS) and factors associated with severe EPS. PLoS One.

[4] Hagan MJ, Shakoor MT (2021). Encapsulating peritoneal sclerosis: imitator of common abdominal disorders. R I Med J.

[5] Vlijm A, Stoker J, Bipat S (2009). Computed tomographic findings characteristic for encapsulating peritoneal sclerosis: a case-control study. Perit Dial Int.

[6] Cornelis T, Oreopoulos DG (2011). Update on potential medical treatments for encapsulating peritoneal sclerosis; human and experimental data. Int Urol Nephrol.

[7] Korte MR, Fieren MW, Sampimon DE, Lingsma HF, Weimar W, Betjes MG (2011). Tamoxifen is associated with lower mortality of encapsulating peritoneal sclerosis: results of the Dutch Multicentre EPS Study. Nephrol Dial Transplant.

[8] Li PK, Chow KM, Cho Y (2022). ISPD peritonitis guideline recommendations: 2022 update on prevention and treatment. Perit Dial Int.

[9] Abidi K, Ferjani M, Jallouli M, Gargah T (2015). Sclerosing encapsulating peritonitis due to peritoneal dialysis successfully treated with tamoxifen. Pediatr Oncall J.

[10] Leventoğlu E, Büyükkaragöz B, Dalgıç A, Fidan K, Söylemezoğlu O, Bakkaloğlu SA (2022). Encapsulated peritoneal sclerosis in an adolescent with kidney transplant after long-term peritoneal dialysis. Exp Clin Transplant.

[11] Awe JA (2013). Abdominal cocoon syndrome (idiopathic sclerosing encapsulating peritonitis): how easy is its diagnosis preoperatively? A case report. Case Rep Surg.

[12] Stefanidis CJ, Shroff R (2014). Encapsulating peritoneal sclerosis in children. Pediatr Nephrol.

[13] Srisajjakul S, Prapaisilp P, Bangchokdee S (2023). Imaging pearls and differential diagnosis of encapsulating peritoneal sclerosis: Emphasis on computed tomography. Clin Imaging.

[14] Kadow JS, Fingerhut CJ, Fernandes Vde B, Coradazzi KR, Silva LM, Penachim TJ (2014). Encapsulating peritonitis: computed tomography and surgical correlation. Radiol Bras.

[15] Guest S (2009). Tamoxifen therapy for encapsulating peritoneal sclerosis: mechanism of action and update on clinical experiences. Perit Dial Int.

